# Clean air in Europe for all: A call for more ambitious action

**DOI:** 10.1097/EE9.0000000000000245

**Published:** 2023-03-09

**Authors:** Hanna Boogaard, Zorana Jovanovic Andersen, Bert Brunekreef, Francesco Forastiere, Bertil Forsberg, Gerard Hoek, Michal Krzyzanowski, Ebba Malmqvist, Mark Nieuwenhuijsen, Barbara Hoffmann

**Affiliations:** a Health Effects Institute, Boston, Massachusetts; b Department of Public Health, Faculty of Health and Medical Sciences, University of Copenhagen, Copenhagen, Denmark; c Institute for Risk Assessment Sciences, Utrecht University, Utrecht, the Netherlands; d Environmental Research Group, School of Public Health, Imperial College, London, United Kingdom; e Department of Public Health and Clinical Medicine, Umeå University, Umeå, Sweden; f Department of Occupational and Environmental Medicine, Faculty of Medicine, Lund University, Lund, Sweden; g ISGlobal, Barcelona, Spain; h Institute for Occupational, Social and Environmental Medicine, Centre for Health and Society, Medical Faculty, University of Düsseldorf, Düsseldorf, Germany

## Introduction

Ambient air pollution is a major global public health risk factor. There is now broad consensus that exposure to air pollution causes an array of adverse health effects based on evidence from a large scientific literature that has grown exponentially since the mid-1990s.^1–4^ Air pollution damages most organ systems and is linked to many debilitating diseases, such as asthma, cardiovascular diseases, chronic obstructive pulmonary disease, pneumonia, stroke, diabetes, lung cancer, and dementia.^5^

The Global Burden of Disease study estimated that in 2019 air pollution ranked as the fourth global risk factor for mortality, surpassed only by high blood pressure, tobacco use, and poor diet.^6^ The European Environment Agency estimated 300,000 premature deaths due to air pollution in the EU-27 in 2020—an unacceptable high air pollution burden.^7^

Air pollution levels have generally declined over the last several decades in Europe, due largely to successful air quality regulation and subsequent improvements in technology and industry. The current air quality legislation in Europe—the Ambient Air Quality Directive (AAQD) from 2008—set limit values for the annual mean of the air pollutants PM_2.5_ and NO_2_ to 25 and 40 µg/m^3^, respectively.^8^ These limit values are criticized for being insufficient to protect the health of EU citizens.^9,10^

The World Health Organization (WHO) released new Air Quality Guidelines (AQG) in September 2021, based on a comprehensive synthesis of the scientific evidence on health effects of air pollution.^4^ They recommended that annual mean concentrations of PM_2.5_ and NO_2_ should not exceed 5 and 10 μg/m^3^, respectively, demonstrating that serious health effects occur above these values. The health community supports full alignment of EU legislation with the 2021 WHO AQG, indicated by a joint statement which was endorsed by more than 140 medical, public health, and scientific societies and patient organizations.^11^

The European Commission (EC) published a proposal to revise the AAQD on October 26, 2022.^12^ The EC also published an accompanying impact assessment, quantifying the expected air pollution concentrations and resulting health- and implementation costs for various policy options.^13^ The European Parliament and the Council are currently considering the proposal. The proposal includes important steps to achieve cleaner air but falls short of what is ultimately needed to maximize public health benefits, for the reasons explained below.

## A clear path toward complete alignment with the 2021 WHO AQG is missing

The proposed new annual limit values are 10 µg/m^3^ for PM_2.5_ and 20 µg/m^3^ for NO_2_ to be met across the EU by 2030. While these proposed limit values are stricter than the current ones, they are still twice as high as the 2021 WHO AQG. Other high-income countries are moving toward more stringent standards. The US Environmental Protection Agency, for example, is considering various alternatives for the current annual PM_2.5_ National Ambient Air Quality Standards (NAAQS) of 12 µg/m^3^ all the way down to 8 µg/m^3^.^14^ The EU may miss the chance to be a role model and a global leader in clean air legislation, if settling for less than full alignment. We call for limit values of 5 and 10 µg/m^3^ for annual PM_2.5_ and NO_2_ by 2030, respectively.

The EC proposal fails to outline a clear pathway toward full alignment with the 2021 WHO AQG even after 2030; instead, it proposes a “regular review mechanism to assure that the latest scientific understanding of air quality guides future decisions.” We underscore that the latest scientific understanding supports full alignment with the 2021 WHO AQG. In addition, multiple options for delaying compliance with limit values will lead to a continued unacceptable high air pollution disease burden in Europe.

## Limit values are needed for ozone

The proposal only provides target values and long-term objectives for ozone, instead of legally binding limit values because of the “complex characteristics of its formation in the atmosphere, which complicate the task of assessing the feasibility of complying with strict limit values.” We disagree with this rationale. Ozone is an important air pollutant, with well-established health effects of short-term (“summer smog”) and long-term exposure and therefore commonly used in air pollution burden assessments.^15^ Ozone levels have decreased much less in the past decades compared to PM_2.5_ and NO_2_, and without reduced emissions of precursors, climate change will increase the concentrations of ozone.^16^ Ozone (as well as other pollutants including PM_2.5_) also increases the heatwave effect on mortality in Europe.^17,18^ We further note that the complexities of ozone formation in the atmosphere are present across the world. Other places, including the United States, do have legally binding air quality standards (NAAQS) for ozone. If the United States can do it, the EU should be able to as well.

Limit values are key to ensure enforceability of the AAQD, as clearly documented by the 2019 Fitness Check conducted by the EC.^19^ We propose the use of binding limit values instead of ineffective target values for ozone, and full alignment with the 2021 WHO AQG, including a long-term (warm season) average of 60 µg/m^3^.

## Adverse health effects of air pollution are underestimated

The systematic reviews underpinning the 2021 WHO AQG were published in 2020 and included health studies available until September 2018.^20,21^ The summary estimates from these global systematic reviews are used in the European impact assessment to calculate the air pollution mortality burden to inform the various policy options and the proposal.

Notably, three very large general population studies funded by the Health Effects Institute were recently published. Those studies examined millions of study participants in the United States, Canada, and Europe and were specifically designed to examine adverse health effects of long-term exposure to low levels of air pollution.^22–24^ All three studies documented associations between mortality and PM_2.5_ concentrations below the current and proposed EU limit values and the US NAAQS. Furthermore, the studies documented either a linear (United States), or a supra-linear (Canada and Europe) exposure-response function between long-term exposure to PM_2.5_ and mortality (supra-linear means a higher effect per additional exposure at low pollutant concentrations than at high concentrations).

The European ELAPSE results are particularly relevant for Europe.^24^ Hence, the EC conducted additional analyses using ELAPSE to estimate the influence of the choice of the exposure-response function on mortality in the impact assessment, partly in response to our suggestions.^25^ The attributable mortality estimates were 40% higher for PM_2.5_ and more than double for NO_2_ using the ELAPSE exposure-response function compared with the estimates from the WHO systematic reviews, all other things kept equal.^13^

Furthermore, only a limited set of morbidity outcomes were included in the impact assessment, and for PM_2.5_ only. Hence, we conclude that the EC impact assessment substantially underestimates the burden of disease due to air pollution. This is especially concerning, as the choice of policy options and the proposed new limit values depended on the net benefit of mitigation actions (benefits from health gains versus costs for implementation of measures).

## Many potential policy options and actions are missing from the feasibility scenario

The maximum technically feasible reduction scenario for projecting future air pollution concentrations considers all available technical measures, irrespective of costs. This assessment shows that, by the year 2030, reaching air pollutant concentration levels that fully align with the 2021 WHO AQG may not be possible for large parts of sampling points in the EU (71% for PM_2.5_).^13^ We note, however, that feasibility is very much related to political will. While technical measures play an important role, other potential realistic abatement options are largely ignored. These include, for example, low or zero emission zones, low-traffic neighborhoods, improving public transportation, promoting active transportation, and incentivizing healthy dietary choices through political action at the production, transport and consumer level. Moreover, accelerated shifts to cleaner fuels and electrification, effective local measures such as coal and biomass combustion bans, and other local or national measures are missing from the feasibility scenario.

The EC conducted a conservative impact assessment that underestimates the health benefits, and one that is based on an incomplete picture of what is possible, leading to a rather unambitious proposal. Despite these shortcomings, the main message of the impact assessment is clear: the health benefits outweigh by far (a factor 6–18) the implementation costs of air quality actions with the largest net benefit (EUR 38 billion) for the policy option of complete alignment with the 2021 WHO AQG by 2030.^13^

## More effort needed to decrease inequalities in health burdens from air pollution

Although air pollution affects all people, certain groups are especially vulnerable and more likely to experience adverse health effects, including pregnant women, children, elderly, chronic disease patients, and those with lower socioeconomic status.^26^ Furthermore, marginalized groups are more likely to live in air pollution hotspot areas, resulting in environmental injustice and additional health disparities.^27,28^ The United States has begun to address the challenges of addressing air pollution-health inequalities through the NAAQS process, where they required additional monitors to be placed in marginalized communities.^14^

We urge the EC to put more emphasis on considering inequalities and recommend using monitoring or modeling to assess whether disparities in exposure are reduced in the future. We note that the current proposed complementary average exposure reduction obligations for long-term PM_2.5_ and NO_2_ will not adequately protect marginalized groups because only urban background monitors are proposed for the calculations. Furthermore, the average exposure is calculated at the regional level (NUTS1 areas), which is too coarse to pick up relevant exposure disparities. We propose that the average exposure reduction obligations should be population-weighted and include hotspot monitor data (e.g., busy roads), ideally augmented with additional monitors to be placed specifically in hotspot areas and in marginalized communities. We recommend using smaller geographical areas (e.g., NUTS2) with clear assignments as to which authorities are responsible for meeting the average exposure reduction obligations, and we emphasize that those obligations should only be complementary to limit values.

## Be wary of the deduction of “natural” source contributions

Similar to the current AAQD, the proposal allows for deductions of “natural” source contributions to exceedances of limit values or exposure reduction obligations. This includes deductions due to wildfires and long-range transported particles from sand and dust storms. Scientific evidence demonstrates that air pollution from those “natural” sources is also harmful to human health, and the vast majority of the health studies underpinning the 2021 WHO AQG do not distinguish between air pollution from “natural” sources versus anthropogenic sources.^4,29,30^ Furthermore, the contribution of “natural” sources is expected to increase due to climate change. We propose to limit the possibility of deduction of “natural” sources.

## Conclusions

We call for more ambition and a clear path in the AAQD toward complete alignment with the 2021 WHO AQG for PM_2.5_, NO_2_ and ozone by 2030. Specifically, we call for limit values of 5 and 10 µg/m^3^ for annual PM_2.5_ and NO_2_, respectively, with the addition of a limit value of 60 µg/m^3^ for long-term (warm season) ozone.

More effort is needed to decrease inequalities in health burdens from air pollution since the current proposed average exposure reduction obligations will not adequately protect marginalized communities. We further caution to be wary of delays in complying with limit values and of the deduction of “natural” source contributions, which are only expected to increase due to climate change.

The robust and stronger associations with mortality and morbidity at very low levels of air pollution underscore the large untapped potential for additional health benefits in Europe and should provide impetus for the European Commission, European Parliament, and the Council to be bold in its ambitions. Moreover, we encourage governments, local authorities and other bodies to continue to exert effort to improve air quality, even if the current and proposed EU limit values have been met.

## Conflicts of interest statement

The authors declare that they have no conflicts of interest with regard to the content of this report.

## Acknowledgments

We thank the ISEE Europe chapter, the ISEE Policy Committee, the ISEE main council, and the ERS Environmental Health Committee for their valuable input.
